# Finite Element Model of Oxygen Transport for the Design of Geometrically Complex Microfluidic Devices Used in Biological Studies

**DOI:** 10.1371/journal.pone.0166289

**Published:** 2016-11-09

**Authors:** Richard J. Sové, Graham M. Fraser, Daniel Goldman, Christopher G. Ellis

**Affiliations:** 1 Department of Medical Biophysics, Western University, London, Ontario, Canada; 2 Cardiovascular Research Group, Division of BioMedical Sciences, Faculty of Medicine, Memorial University, St. John’s, Newfoundland, Canada; Bioinformatics Institute, SINGAPORE

## Abstract

Red blood cells play a crucial role in the local regulation of oxygen supply in the microcirculation through the oxygen dependent release of ATP. Since red blood cells serve as an oxygen sensor for the circulatory system, the dynamics of ATP release determine the effectiveness of red blood cells to relate the oxygen levels to the vessels. Previous work has focused on the feasibility of developing a microfluidic system to measure the dynamics of ATP release. The objective was to determine if a steep oxygen gradient could be developed in the channel to cause a rapid decrease in hemoglobin oxygen saturation in order to measure the corresponding levels of ATP released from the red blood cells. In the present study, oxygen transport simulations were used to optimize the geometric design parameters for a similar system which is easier to fabricate. The system is composed of a microfluidic device stacked on top of a large, gas impermeable flow channel with a hole to allow gas exchange. The microfluidic device is fabricated using soft lithography in polydimethyl-siloxane, an oxygen permeable material. Our objective is twofold: (1) optimize the parameters of our system and (2) develop a method to assess the oxygen distribution in complex 3D microfluidic device geometries. 3D simulations of oxygen transport were performed to simulate oxygen distribution throughout the device. The simulations demonstrate that microfluidic device geometry plays a critical role in molecule exchange, for instance, changing the orientation of the short wide microfluidic channel results in a 97.17% increase in oxygen exchange. Since microfluidic devices have become a more prominent tool in biological studies, understanding the transport of oxygen and other biological molecules in microfluidic devices is critical for maintaining a physiologically relevant environment. We have also demonstrated a method to assess oxygen levels in geometrically complex microfluidic devices.

## Introduction

Red blood cells (RBCs) have been shown to release adenosine triphosphate (ATP) in response to numerous stimuli [1–4], including hemoglobin oxygen saturation (SO_2_) [5]. Following release, ATP binds to purinergic receptors on capillary endothelial cells which conduct an electrical response to upstream arterioles, leading to their vasodilation [6]. Therefore, RBCs are believed to play an important role in the local regulation of oxygen (O_2_) distribution through the SO_2_ dependent release of ATP [7, 8].

In addition to its importance in regulatory physiology, SO_2_ dependent ATP release has been shown to be impaired in many cardiovascular diseases such as sepsis [9], prediabetes [10] and type II diabetes [11]. In these studies, the amount of ATP released was decreased for the same stimulus. Therefore, RBCs become a potential screening target for cardiovascular disorders.

Although SO_2_ dependent ATP release has been measured, there are currently no studies that quantify the dynamics of this process. Since ATP release is believed to be involved in the regulation of O_2_ distribution, understanding the dynamics is crucial for our understanding of the regulatory pathway. The time required for ATP to be released determines the spatial sensitivity for the RBC to signal the endothelium changes in their SO_2_.

The ultimate goal of our research is to develop a cost effective tool to quantify the dynamics of ATP release from RBCs furthering our ability to characterize the underlying physiology of blood flow regulation. Various studies in the literature have developed means of controlling O_2_ in microfluidic devices for a variety of applications [12–19], e.g. microfluidic devices for establishing hypoxia in cell cultures [12]. Many of these studies apply mathematical modelling to verify that they are correctly maintaining their target O_2_ levels [13–15, 17, 18].

In an earlier study, we employed a novel micro-delivery approach to change local oxygen levels *in vivo* [20, 21]. We also previously described a computational model of an idealized microfluidic device to measure the dynamics of SO_2_ dependent ATP release *in vitro* [22]. The objective of the design was to create a spatial step change in SO_2_ in a steady flowing channel, then measure the corresponding ATP released from the RBCs. The resulting spatial information can then be translated into temporal. This approach was adapted from *Wan et al* [23], and is described in detail in our previous study [22]. In contrast to other devices for controlling oxygen, our application is intended to spatially control the O_2_ content of flowing RBCs.

Although the previous model predicted that the device was able to create a sufficient drop in O_2_, the idealized microfluidic device was not practical to fabricate using common soft lithography techniques. This motivated a new device design that is both practical and functional. However, the complex geometric design of the new device and the use of O_2_ permeable materials makes predicting the SO_2_ of the RBCs more difficult. Therefore, in this study we develop a 3D computational model of the new device, in order to optimize the dimensions, and to ensure that O_2_ characteristics in the device are sufficient for quantifying the dynamics of SO_2_ dependent ATP release. Additionally, this model will be crucial to aid in the analysis of results of subsequent studies using this design.

## Methods

**Fig 1:**
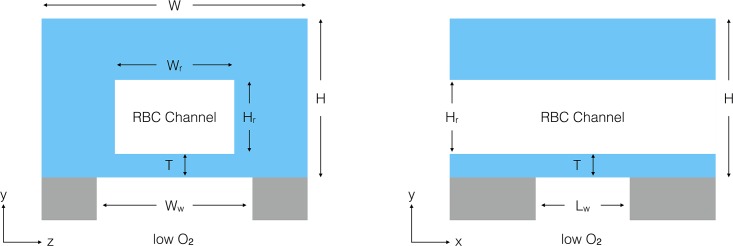
Device Design. Diagram of x-normal (left) and z-normal (right) views of the RBC channel and exchange window. The light blue colour represents PDMS and the grey colour represents glass.

In this work, we take a computational approach to design a device for measuring the dynamics of SO_2_ dependent ATP release from RBCs. We propose a device that consists of two parts (see Fig 1), the first part being a microfluidic channel fabricated in PDMS using soft lithography techniques. This channel will be embedded in PDMS using a mould, and sealed with a PDMS spin coating technique. The second part being a large oxygen impermeable gas flow channel with a window to allow gas exchange between the two channels. The two parts are aligned orthogonal to each other with the gas exchange window centred at their intersection. The bottom channel is designed to deliver a gas with a low concentration of oxygen, whereas the top microfluidic channel will deliver fully oxygen-saturated red blood cells suspended in a physiological buffer. The large gradient in oxygen partial pressure at the exchange window between the two channels drives the desaturation of the RBCs.

In the present work, we will explore the influence of geometry on the device’s ability to control the oxygen levels the cells are exposed to using a computational model of fluid dynamics and mass transfer (kinetics of ATP release and reaction with luciferin/luciferase will be simulated in a subsequent model); see Fig 1 for the geometry. Due to the large number of geometric parameters we can vary, we start by analyzing a 1D analytic model of oxygen exchange to guide our choice of parameters to vary with the 3D model.

### Analytic Model

The following equation describes the oxygen partial pressure, *PO*_2_, of the blood in a microfluidic device with an exchange window of length *L*_*w*_ centred at *x* = 0.

$$D\frac{d^{2}PO_{2}}{dx^{2}}-c\frac{dPO_{2}}{dx}=\left\{\begin{array}{cc} k_{1}\left(PO_{2}-P_{0}\right), & x\in \left(-\infty ,-\frac{L_{w}}{2}\right)\\ k_{1}\left(PO_{2}-P_{0}\right)+k_{2}\left(PO_{2}-P_{l}\right), & x\in \left[-\frac{L_{w}}{2},\frac{L_{w}}{2}\right]\\ k_{1}\left(PO_{2}-P_{0}\right), & x\in \left(\frac{L_{w}}{2},\infty \right) \end{array}\right.$$ (1)

with boundary conditions,

$$\lim_{x\to -\infty }P\left(x\right)=P_{0}$$ (2a)

$$\lim_{x\to \infty }P\left(x\right)=P_{0}$$ (2b)

where *D* is diffusivity of oxygen in plasma, *c* is the flow velocity, and *k*_1_ and *k*_2_ are the rates of oxygen permeation through the walls of the channel and the exchange window respectively; they depend on the thickness of the PDMS walls and the permeability of PDMS to O_2_. *P*_0_ is the external oxygen partial pressure and *P*_*l*_ is the oxygen partial pressure of the gas in the exchange window. The microfluidic device is assumed to be infinitely long so that we can neglect inlet/outlet effects and the effects of oxygen binding to hemoglobin are neglected in the model.

#### Non-Dimensional Analysis

**Fig 2:**
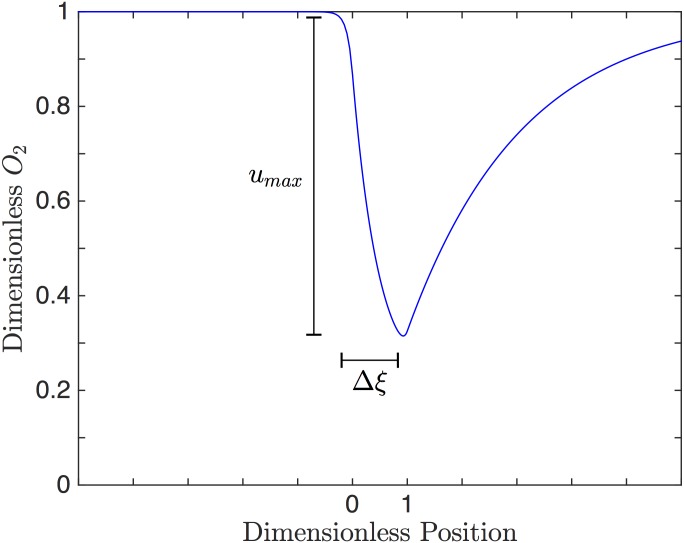
Dimensionless Solution. Solution to the 1D dimensionless model. The dimensionless O_2_, *v*, is shown as a function of dimensionless position, *ξ* for *Pe* = 10, *S*_1_ = 5 and *S*_2_ = 20. The exchange window is between 0 and 1. The red arrow indicates Δ*ξ* = 1.04 with *u*_*max*_ of 0.68. For this set of conditions, dimensionless O_2_ begins to fall just before the window and continues to fall across the length of the window before beginning to rise.

Non-dimensionalization allows us to determine the parameters that effect the behaviour of the solution. We introduce the following dimensionless parameters: $\xi =\frac{x}{L_{w}}+\frac{1}{2}$, $v=\frac{PO_{2}-P_{l}}{P_{0}-P_{l}}$, *u* = 1 − *v*, $Pe=\frac{cL_{w}}{D}$, $S_{1}=\frac{k_{1}L_{w}^{2}}{D}$ and $S_{2}=\frac{k_{2}L_{w}^{2}}{D}$ which gives,

$$\frac{d^{2}u}{d\xi^{2}}-Pe\frac{du}{d\xi }-S_{1}u=\left\{\begin{array}{cc} 0, & \xi \in (-\infty ,0)\\ S_{2}\left(u+1\right), & \xi \in [0,1]\\ 0, & \xi \in (1,\infty ) \end{array}\right.$$ (3)

with homogeneous boundary conditions at infinity. Dimensionless O_2_ is represented by *v* and the dimensionless drop in O_2_ is represented by *u*. From the non-dimensionalization, we see that the behaviour of the solution depends on three independent parameters, *Pe*, *S*_1_ and *S*_2_. *Pe* is the Peclet number and represents the ratio of convective to diffusive transport. *S*_1_ and *S*_2_ are dimensionless *k*_1_ and *k*_2_, respectively. The dimensionless solution for a specific case is shown in Fig 2.

There are three main criteria that we will use to assess the performance of the device. First, the device must be able to cause a large enough change in O_2_ to elicit ATP release from the RBCs. This first criterion can be quantified by taking the maximum drop in PO_2_. Eq 4 gives this criterion in terms of the 1D model parameters,

$$\Delta PO_{2_{max}}=\left(P_{0}-P_{l}\right)u_{max}$$ (4)

where *u*_*max*_ is the maximum value of *u*(*ξ*).

Second, the drop in O_2_ must be sufficiently rapid to resolve the dynamics of ATP release. This can be quantified as the amount of time between the maximum and the minimum PO_2_. Since this time depends on Δ*PO*_2_*max*__, we normalize it with respect the drop. This criterion is a representation of the rate of O_2_ drop. Eq 5 gives the second criterion in terms of the 1D model parameters,

$$\frac{\Delta PO_{2_{max}}}{\Delta t}=\frac{c(P_{0}-Pl)}{L}\frac{u_{max}}{\Delta \xi }$$ (5)

where Δ*ξ* is the change in dimensionless position between when the maximum and minimum value of *u*(*ξ*) deviate by 1% of their original values.

Third, the temporal resolution of the system has to be able to resolve the dynamics. This can be quantified in terms of the spatial resolution, *ϵ*, of the system and the flow velocity, c, as $\tau =\frac{\epsilon }{c}$.

#### Parameter Selection

**Fig 3:**
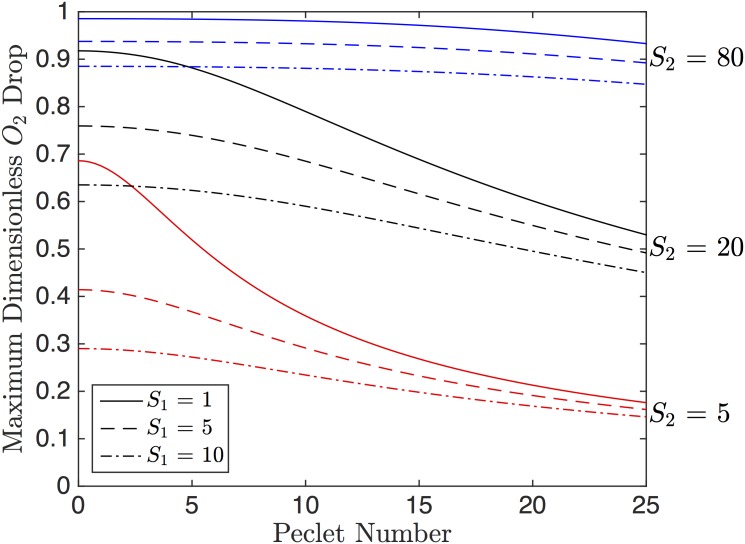
Dimensionless O_2_ Drop. The maximum dimensionless O_2_ drop, *u*_*max*_, as a function of Péclet number, *Pe*. The solid, dashed and dot-dashed curves represent *S*_1_ values of 1, 5 and 10, respectively. The red, black and blue curves represent values of *S*_2_ of 5, 20 and 80 respectively.

To select the ideal parameters for the 1D model, we optimize our three performance criteria. The parameters involved are the physical parameters (*P*_0_ − *P*_*l*_), *c*, and *L* and the dimensionless parameters *u*_*max*_ and Δ*ξ*, which are dependent on *Pe*, *S*_1_ and *S*_2_. In order to maximize the maximum drop in *PO*_2_, *u*_*max*_ has to be maximized. Fig 3 shows *u*_*max*_ as a function of *Pe* for different values of *S*_1_ and *S*_2_. From Fig 3, *u*_*max*_ is maximized as *Pe* → 0, *S*_1_ → 0 and *S*_2_ → ∞. As *S*_2_ increases, *u*_*max*_ becomes less sensitive to *S*_1_ and *Pe*.

**Fig 4:**
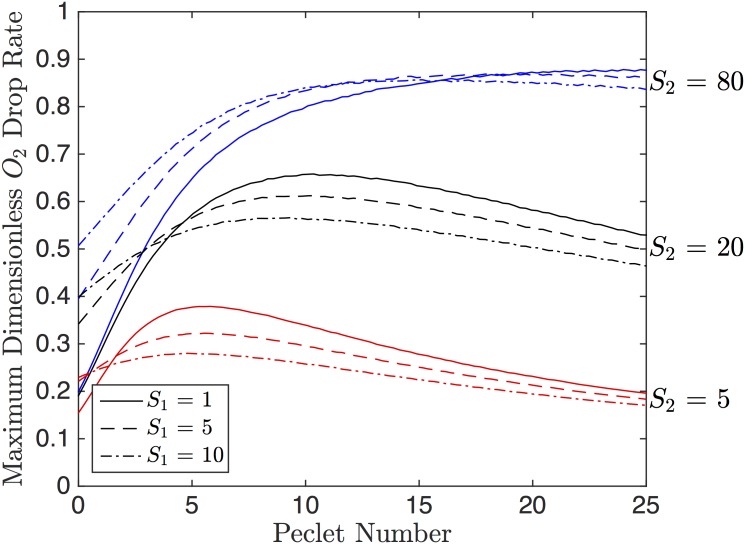
Dimensionless Drop Rate. The dimensionless rate of O_2_, *u*_*max*_/Δ*ξ*, as a function of the Péclet number, *Pe*. The solid, dashed and dot-dashed curves represent *S*_1_ values of 1, 5 and 10, respectively. The red, black and blue curves represent values of *S*_2_ of 5, 20 and 80 respectively.

In order to maximize the rate of O_2_ drop, *u*_*max*_/Δ*ξ* should be maximized. Fig 4 shows *u*_*max*_/Δ*ξ* as a function of *Pe* for varying values of *S*_1_ and *S*_2_. From Fig 4, *u*_*max*_/Δ*ξ* is maximized for large *Pe*, small *S*_1_, and large *S*_2_. The effect of *S*_2_ on O_2_ drop rate is larger than that of *S*_1_.

To optimize both *u*_*max*_ and *u*_*max*_/Δ*ξ* a compromise in *Pe* must be chosen since *u*_*max*_ is maximized for small *Pe* and *u*_*max*_/Δ*ξ* is maximized for large *Pe*. However, since the effects of *Pe* on *u*_*max*_ are small for large *S*_2_, if we can choose large *S*_2_, we can choose the Pe to satisfy the optimization of *u*_*max*_/Δ*ξ*.

Since we cannot control diffusivity, the dimensionless parameters must be controlled by altering *c*, *L*, *k*_1_ and *k*_2_. To minimize *S*_1_, *k*_1_ should be as small as practical; physically this can be achieved by making the walls as thick as possible. To maximize *S*_2_, *k*_2_ should be made as large as possible; this can be achieved by making the spin coat layer as small as possible. (*P*_0_ − *P*_*l*_) should also be made as large as practical.

The flow velocity, *c*, affects *Pe*, Δ*PO*_2_*max*__/Δ*t* and the temporal resolution, *τ*. All three parameters are optimized with a larger *c*. The effect of window length, *L*_*w*_, is less straightforward since it effects the dimensionless parameters and Δ*PO*_2_*max*__/Δ*t*. *S*_1_ and *S*_2_ are sensitive to *L*_*w*_ since they are related quadratically, and since they have opposite optima, care must be taken while choosing the length of the exchange window. The effect of the window length will be explored with the 3D model.

### Computational Model

#### Mesh Generation

The geometry construction and mesh generation were performed using the open source software GMSH [24]. The same mesh was used for both the fluid dynamics simulations and the mass transfer simulations.

For each geometry, a hybrid mesh was used, with structured regions in the flow channels and an unstructured region in the PDMS area (which is far from the region of interest) to reduce the number of elements. Element sizes were decreased until the solution no longer changed with mesh resolution. The resulting meshes varied between simulations, but typically the smallest elements (located in the RBC channel) were on the order of 0.01 mm, and the largest elements (located in the PDMS) were 0.5–1.0 mm.

#### Fluid Dynamics Simulations

The flow in both channels were assumed to be Newtonian, incompressible and isothermal. The blood was assumed to be a single homogeneous fluid. The resulting steady-state equations for flow in both channels are given by:

$$\nabla \cdot {\vec{v}}_{i}=0$$ (6a)

$$\rho_{i}{\vec{v}}_{i}\cdot \nabla {\vec{v}}_{i}=\mu_{i}\nabla^{2}{\vec{v}}_{i}-\nabla p$$ (6b)

where ${\vec{v}}_{i}$, *ρ*_*i*_, *μ*_*i*_ are the velocity, density and dynamic viscosity of the fluid respectively and *p* is the hydrodynamic pressure in the fluid. The subscripts *i* = *g*, *p*, *rbc* refer to the gas, plasma and red blood cells. Since the blood is assumed to be homogeneous ${\vec{v}}_{p}={\vec{v}}_{rbc}$, *ρ*_*p*_ = *ρ*_*rbc*_ and *μ*_*p*_ = *μ*_*rbc*_.

Eqs 6a and 6b were solved numerically using open source software OpenFOAM [25]. The software discretizes the equations using a finite volume method and solves the coupled system of equations using the Semi-Implicit Method for Pressure Linked Equations [26].

#### Mass Transfer Simulations

The following equations were used to model the oxygen transport throughout the microfluidic device [27]:

$$D_{pdms}k_{pdms}\nabla^{2}PO_{2}=0\;\;\;\;\;\;\;\;\in \Omega_{pdms}$$ (7a)

$$k_{g}{\vec{v}}_{g}\cdot \nabla PO_{2}=D_{g}k_{g}\nabla^{2}PO_{2}\;\;\;\;\;\;\;\;\in \Omega_{g}$$ (7b)

$$\left[\left(1-Ht\right)k_{p}{\vec{v}}_{p}+Ht\left(k_{rbc}+\left[Hb_{T}\right]\frac{dSO_{2}}{dPO_{2}}\right){\vec{v}}_{rbc}\right]\cdot \nabla PO_{2}=D_{p}k_{p}\nabla^{2}PO_{2}\;\;\;\;\;\;\;\;\in \Omega_{rbc}$$ (7c)

where *D*_*i*_, *k*_*i*_, *PO*_2_, *Ht*, and [*Hb*_*T*_] are the diffusivity, solubility, oxygen partial pressure, hematocrit and total heme concentration of the blood respectively. The different regions of the device are designated by *Ω*_*j*_, where the subscripts *j* = *pdms*, *g*, *rbc*, *p* represent the PDMS, gas, red blood cells and plasma respectively. The derivative $\frac{dSO_{2}}{dPO_{2}}$ can be found from the Hill equation [28]:

$$SO_{2}=\frac{PO_{2}^{N}}{P_{50}^{N}+PO_{2}^{N}}$$ (8)

where *SO*_2_ is hemoglobin oxygen saturation, *N* is the Hill coefficient and *P*_50_ is the partial pressure of oxygen at 50% saturation.

Eqs 7a–7c were discretized using in-house software that utilizes a stabilized Galerkin finite element method programmed in C++; a least squares stabilizer was used in the convective regions of the domain [29]. The resulting non-linear system of equations were iteratively linearized and solved with the Generalized Minimal Residual Method [30]. Convergence was verified for the extreme cases by refining the mesh to be sure that the solution was independent of the choice of discretization. All simulations were run on a personal computer with an 8 core 4.2 GHz processor with 16 GB of RAM. Simulation times were less than an hour on one core.

To quantify the *O*_2_ exchange in the 3D model we define the weighted drop in *PO*_2_ by the following,

$$WD=max_{PO_{2}\in x}\frac{1}{W_{r}}\int_{z=-\frac{W_{r}}{2}}^{z=\frac{W_{r}}{2}}\int_{y=y_{0}}^{y=y_{0}+H_{r}}PO_{2}\left(x,y,z\right)e^{-\mu (y-y_{0})}dydz$$ (9)

where *W*_*r*_ and *H*_*r*_ are the width and height of the RBC channel, respectively, *y*_0_ is the location of the bottom of the channel and *μ* is the optical attenuation of blood plasma. This is a weighted integral of the PO_2_ along the depth of the channel (y-direction) to give a stronger weighting to the PO_2_ values closer to the bottom of the channel since that is where the detector will be located. It is then averaged across the width of the channel (z-direction) and the maximum value is taken along the stream-wise direction (x-direction).

The spatial drop time is quantified in the same way as for the 1D model; the 3D profile is reduced to one dimension by using the same weighted integral in y and taking the centreline in z.

## Results

**Fig 5:**
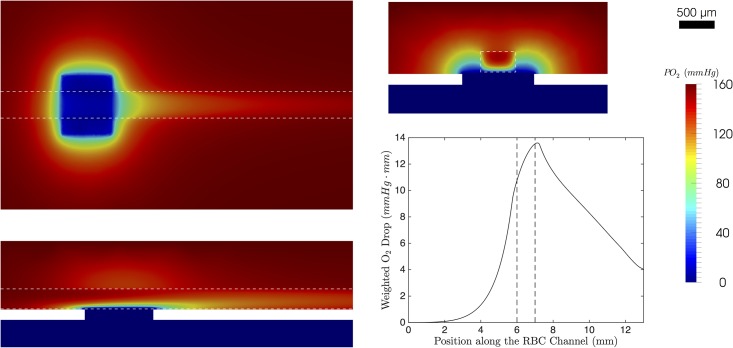
Solution to the 3D model. The top left colormap shows the y-normal plane close to the bottom edge of the channel (y = 0.5775). The bottom left colormap shows the z-normal plane through the center of the geometry. The colormap on the top right shows the x-normal plane through the center of the geometry. The white dashed line indicates the RBC channel. The plot in the bottom right shows the weighted centreline drop in PO_2_. The black dashed line indicates the location of the exchange window.

To investigate the effects of 3D geometric features, we simulated the O_2_ transport in the microfluidic device for various dimensions of the RBC channel. In particular, we varied cross-sectional area of the RBC channel, as well as its aspect ratio to capture the effects of channel cross-section. In addition, we varied the length of the exchange window to determine how the O_2_ drop and its rate are affected. We also varied spin-coat thickness to determine how important it is for our particular geometry and physical conditions. Fig 5 shows a specific solution of the 3D model using the parameters in Table 1; this figure shows the weighted centreline profile.

**Table 1:** Model Parameters.

Parameter	Value
Baseline Oxygen Partial Pressure (*mmHg*)	160
Gas Oxygen Partial Pressure (*mmHg*)	0
Diffusivity of Oxygen in Nitrogen (*mm*^2^/*s*)	17.6
Solubility of Oxygen in Nitrogen (*μM*/*mmHg*)	5.342105
Solubility of Oyxgen in RBCs (*μM*/*mmHg*)	1.47
Diffusivity of Oxygen in Plasma (*mm*^2^/*s*)	0.00275
Solubility of Oxygen in Plasma (*μM*/*mmHg*)	1.33
Diffusivity of Oxygen in PDMS (*mm*^2^/*s*)	0.00355
Solubility of Oxygen in PDMS (*μM*/*mmHg*)	17.959
Total Heme Concentration (*μM*)	5350
Oxygen Partial Pressure at 50% Saturation (mmHg)	37
Hill Coefficient	2.7
Hematocrit	0.1

**Fig 6:**
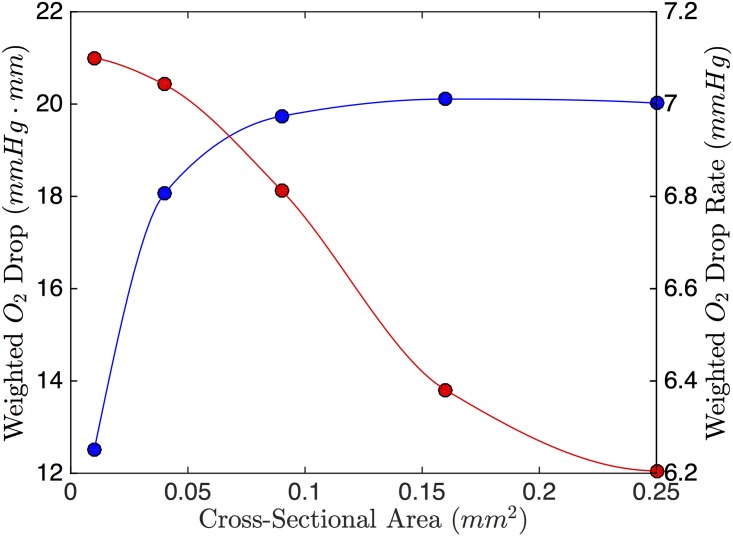
Effect of RBC Channel Cross Sectional Area. The weighted O_2_ drop (blue) and weighted O_2_ drop rate (red) as a function of RBC channel cross-sectional Area. The channels simulated are square in cross-section.

First, the cross-sectional area of the RBC channel was varied maintaining a constant mean velocity. The channels simulated were square in cross-section ranging from 100x100 *μm*^2^ to 500x500 *μm*^2^. The maximum drop in PO_2_ increased with increasing cross-sectional area (see Fig 6). This appears to be due to the increased surface area for exchange as well as the increased volume in the channel contributing to the weighted drop. In contrast, rate of drop decreases with area. This is likely due to the increasing volume of the RBC channel.

**Fig 7:**
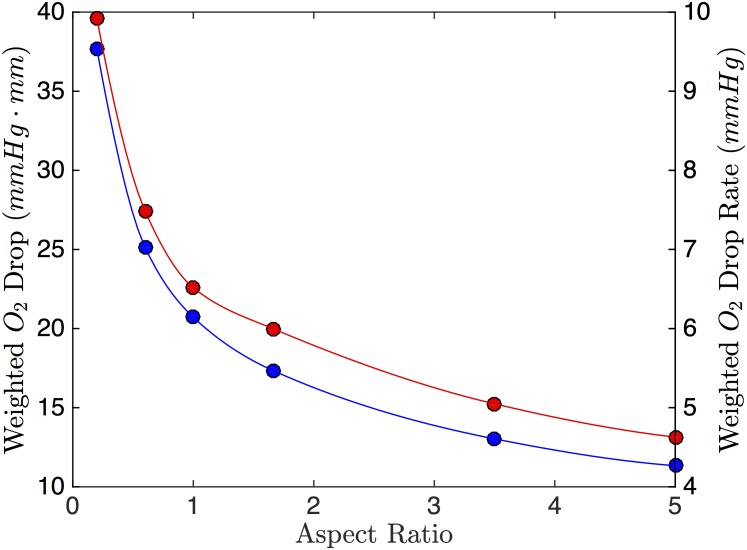
Effect of RBC Channel Aspect Ratio. The weighted O_2_ drop (blue) and weighted O_2_ drop rate (red) as a function of RBC channel aspect ratio (width:height). The area of the channels simulated was held constant (0.15 mm^2^).

**Fig 8:**
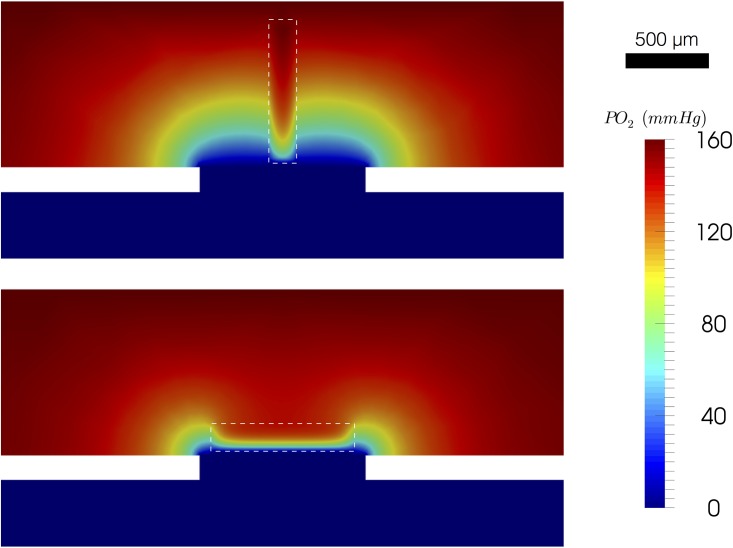
Simulation of RBC Channel Aspect Ratios. The PO_2_ solution showing the x-normal plane through the center of the geometry for two different aspect ratios. The colormap on the top shows the simulation results for a channel with 1:5 width to height ratio. The colormap on the bottom shows the simulation results for a channel with a 5:1 width to height ratio. The white dashed line indicates the RBC channel.

From Fig 7, channels will smaller height to width ratio perform better at dropping the O_2_ compared to short, wide channels of the same area. This is due to multiple factors: first, the channels that are taller have more volume contributing to the weighted drop (see Eq 9). Second, the taller channels are thin, allowing the more surface area on their sides to be exposed to low O_2_. Fig 8 shows colour maps for the two extreme cases. The change in drop rate follows the same trend as with the weighted drop.

**Fig 9:**
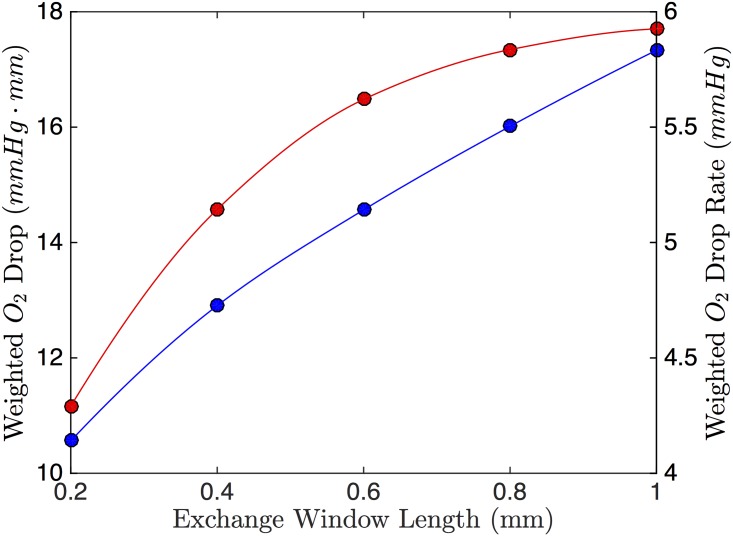
Effect of Exchange Window Length. The weighted O_2_ drop (blue) and weighted O_2_ drop rate (red) as a function of exchange window length.

Increasing the length of the window causes an increased drop in PO_2_ and an increased drop rate (see Fig 9). The increase in PO_2_ drop can be explained by having a longer window, exposing more of the channel to low O_2_.

**Fig 10:**
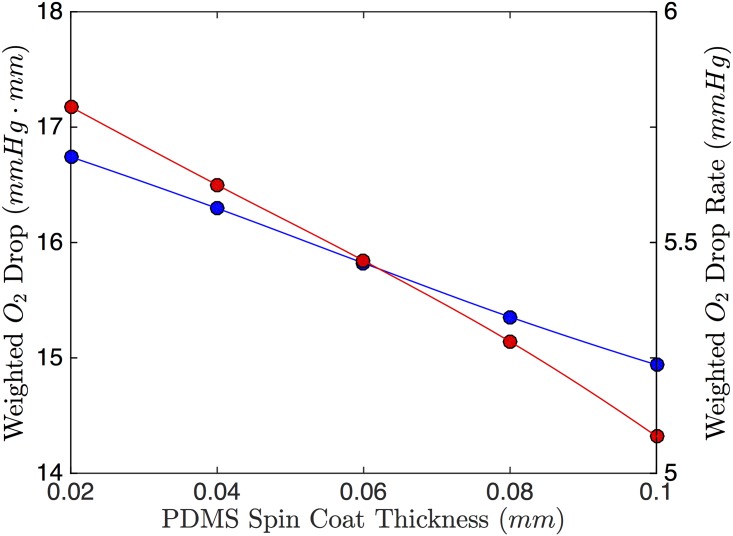
Effect of Spin Coat Thickness. The weighted O_2_ drop (blue) and weighted O_2_ drop rate (red) as a function of PDMS spin-coat thickness.

**Fig 11:**
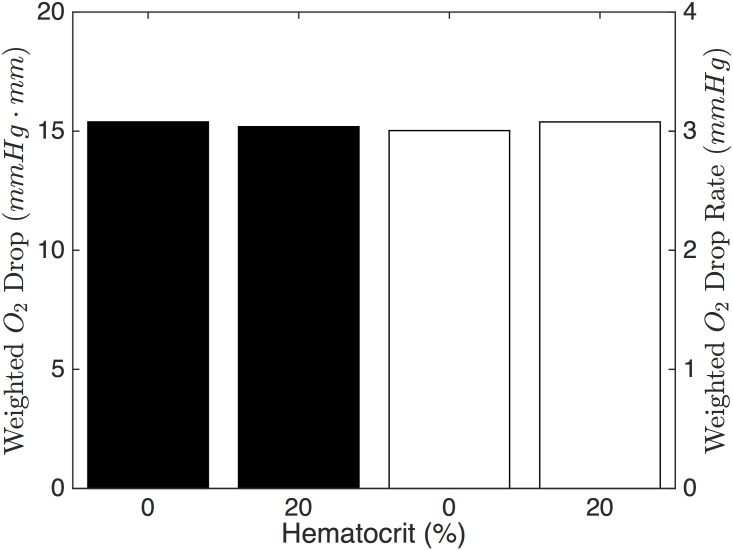
Effect of Hematocrit. The weighted O_2_ drop (black bars) and weighted O_2_ drop rate (white bars) for 0% and 20% hematocrit. A negligible effect is shown for both.

Increasing the spin-coat layer thickness causes a decrease in the drop in PO_2_; this relationship is approximately linear. The change in drop rate follows a similar trend to the drop in O_2_ (see Fig 10). In terms of the 1D model, increasing the spin-coat thickness increases *S*_2_, which causes a smaller drop in O_2_ and a smaller drop rate (see Figs 3 and 4); this trend agrees with our 3D results. Hematocrit has negligible effect on both the weighted drop and the drop rate (Fig 11).

## Discussion

Ellsworth *et al* suggested that ATP release time is less than 500 milliseconds [31] based on the RBC transit time in an isolated arteriole preparation exposed to low oxygen levels. Based on this estimate, we require a system with a resolution on the order of milliseconds. Wan et al measured shear dependent ATP release time and reports it to be on the order of 25–75 milliseconds [23]. If the mechanisms responsible for ATP release are similar for both shear-dependent and O_2_ dependent release, then we expect, ATP release time to be similar to the value measured in their study. Understanding the time course for ATP release from RBCs is important since it determines where in the vasculature ATP is released and where the vessels will sense ATP. Further, many inflammatory diseases have been associated with impaired ATP release, thus a measurement of ATP release dynamics could be used to screen for these diseases. ATP believed to be responsible for local control of oxygen, if release time is delayed, then this will impair ability to locally control oxygen; could become potential pharmaceutical target.

In previous work, we showed the feasibility of using steady-state flow to measure the dynamics of SO_2_-dependent ATP release *in vitro* [22]. This work was based on an ideal device in order to test the viability of the concept. We demonstrated two important design criteria, first, that the device was able to cause a sufficient drop in O_2_ and second, that we were able to recover time-dependent changes in the ATP signal. Although this study was able to show the feasibility of our device, the idealized design described in this article was not practical to fabricate.

**Fig 12:**
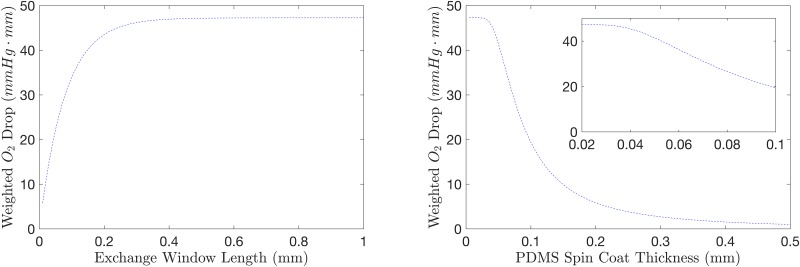
1D Model Prediction of Geometric Parameters. Weighted O_2_ drop was simulated using the 1D model to determine the effect of varying exchange window length (left) and PDMS spin-coat thickness (right). The inset in the right panel shows the parameter range used in the 3D simulations. The results of the simulations were re-dimensionlized in order to be comparable to the 3D simulation results (see Figs 9 and 10). Note: the parameters range used in the 3D simulations for the exchange window length was 0.1–1.

With the practical considerations in mind, we developed a simple 1D model of oxygen transport in our device in order to qualitatively evaluate some of the simulation parameters to reduce the number of simulations required to determine the optimal design for our application. The 1D model is an idealization of the device and does not account for geometric information such as the RBC channel’s cross-section dimensions, though some of the geometric information is embedded in the model parameters, such as PDMS thickness. To compare the performance of the 1D model against our 3D simulations, we show the weighted O_2_ drop predicted by the 1D model for varying exchange window lengths and PDMS spin coat thicknesses (see Fig 12).

The 1D model predicts the O_2_ drop to level off and reach a steady value as the exchange window length increases. The 3D model predicts the O_2_ drop to increase with increasing window length, though for the parameters used, the simulation results do not reach a steady drop. Further, the value of the drop is considerably lower then predicted by the 1D model. Considering the PDMS spin coat thickness, the 1D model predicts the O_2_ drop to approach zero as the thickness increases; the O_2_ drop is predicted to approach the maximum drop as the thickness approaches zero. Comparing these results to the 3D simulations, the O_2_ drop is substantially lower than predicted by the 1D model and does not begin to level off for the lowest thickness simulated. While the 1D model predictions appear reasonable, the O_2_ drop is overestimated compared to the 3D model and is more sensitive to the variations in the geometric parameters.

The 1D model approaches the 3D model as the RBC channel becomes infinitely wide and its height becomes infinitesimally small. It assumes cross-stream diffusion in the RBC flow is negligible and diffusion in the PDMS occurs only vertically. Further, the 1D model also assumes that the fluid velocity in the gas channel is infinite, so that the low oxygen is maintained at the gas channel side of the exchange surface. Due to these simplifications, the 1D model overestimates the drop in O_2_; these simplifications also account for the increased sensitivity of the geometric parameters. Although the 1D model does not accurately account for the O_2_ content in the device, it allows us to qualitatively determine the behaviour of changing certain parameters. Also, since the model can be solved analytically, we can simulate a large range and achieve practically continuous information. Therefore, the 1D analytical model is a useful tool for qualitatively understanding the physical details of our system, but a 3D model is required to quantify the extent of the behaviour.

Though the 1D model was useful in helping us determine how the O_2_ permeable walls will affect the O_2_ exchange, it fails to capture some of the important 3D features. Thus, we used a 3D model to explore how the O_2_ exchange is affected when we have a channel of finite cross-section and diffusion around the sides of the RBC channel from the exchange window and outside surfaces.

The first 3D aspect of the device geometry we look at is the RBC channel’s cross-section. From Fig 6, it is clear that there is an optimal cross-sectional area. Channels with larger cross-sectional areas have larger surface areas, leading to more area for the exchange. However, the exchange is limited to the lower walls of the channel since the exchange window is on the bottom. For the larger channels, the width of the window limits how much of the side walls’ surface area is exposed to the low levels of O_2_. If we now allow the channel to increase in surface area, but not get any wider, we can optimize the amount of surface area exposed to the low levels of oxygen; this is demonstrated in Figs 7 and 8. In contrast our previous results showed channels that are less deep are more beneficial since the drop extends across the whole channel [22]. However, this was because the 2D model in [22] assumes that the channel and window are infinitely wide; thus, it neglects the effect of low oxygen on the sides of the channel.

In the 1D model, the length of the window is the characteristic length. Thus it not only effects O_2_ exchange through Eq 5, but it also effects *u*_*max*_ and *u*_*max*_/Δ*ξ* through the dimensionless parameters. From Fig 9, we see that having a longer window is better for both the drop and the drop rate of O_2_. The benefits of longer windows will become less important as O_2_ in the channel approaches zero. Practically, a very large exchange window will result in a structurally weak device; the thin PDMS layer separating the RBC and gas channel may rupture or deform. Therefore, the exchange window should be large enough to cause a sufficient drop in oxygen, but not so long that the device becomes structurally compromised.

The 1D model indicates that the ideal device would have an infinitely thin spin-coat layer. However, practically, we need a barrier that closes the RBC channel so that it does not leak; thus it has to be thick enough so that it does not rupture under the pressure of the flow. Physically, we can fabricate spin-coat thicknesses on the order of 20 *μ*m and these channels do not rupture under the pressure of the flow. From Fig 10, we can see that in the range of practical spin-coat sizes (20–100 *μ*m), the spin-coat thickness does not affect the O_2_ exchange substantially. Therefore, small variability in the thickness of the spin coat layer will not affect the effectiveness of the device.

Interestingly, hematocrit has only a small effect on the O_2_ exchange in our device. From Fig 11, we can see that lower hematocrit is better for exchange, though, the change is not large. An important point to consider is that larger hematocrit will result in more RBCs available to release ATP, increasing the amount of signal coming form the system. However, the amount of RBCs affects the optics of the system since they absorb visible light. Therefore, we should use a hematocrit that is large enough to attain a measurable ATP signal, but not so large that all the signal is attenuated by the cells. We considered this effect in a previous study [22].

In this study, we did not simulate every possible geometric parameter, such as the length of the RBC channel, the height of the PDMS around the channel, the width of the window, and the dimensions of the gas channel. We no not expect the length of the RBC channel to be too important for exchange. For the purposes of the analysis of the experimental results, the flow should be fully developed. Therefore the channel should be longer than the entrance length, though at the flow rates typically used in microfluidics, the entrance lengths are quite small (on the order of 3–30 *μ*m). As for oxygen exchange in shorter channels, the drop will be smaller since the source of oxygen is closer to the exchange window. This will also result in a steeper rate. However, these effects are only important for channels on the order of the entrance length.

Practically, the exchange window cannot exceed a width of 1 mm due to the risk of the PDMS spin-coat deforming into the window causing distortion to the optical image. Intuitively, wider windows will improve exchange since there will be more area for exchange, though the effect will become insignificant as the window becomes much wider than the channel. From the 1D model, we expect more PDMS around the RBC channel to be better as this decreases the effective permeability of the walls. However, if there is too much PDMS above the channel, there may be optical problems.

**Fig 13:**
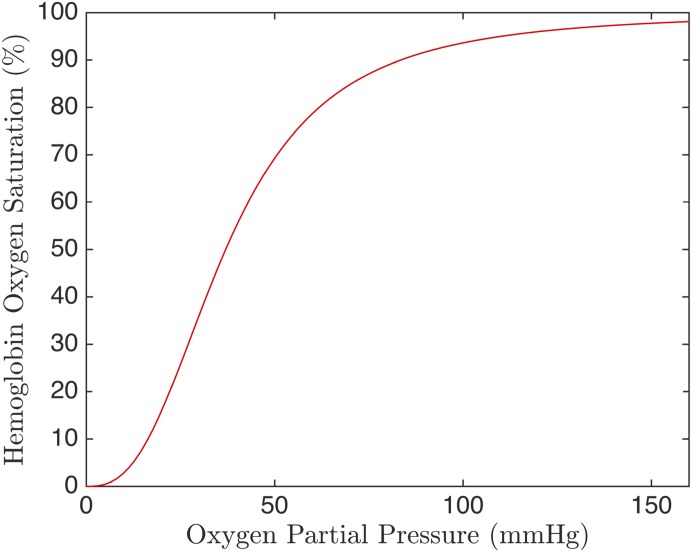
Hemoglobin Binding Curve. Hemoglobin oxygen saturation as a function of partial pressure determined by the Hill equation.

This study focuses on the ability of the proposed microfluidic device to cause a drop of PO_2_ in a flowing RBC suspension. However, the main goal of this device is to measure the release of ATP from RBCs in response to their SO_2_. The relationship between PO_2_ and SO_2_ is modelled by the Hill equations (Eq 8). Fig 13 shows the Hill equation for human RBCs. This figure demonstrates that for low and high PO_2_, large changes in PO_2_ are required to change saturation. In contrast, in the mid-range of PO_2_, only small changes in PO_2_ are required to produce large changes in SO_2_. Since the RBCs in our simulation span PO_2_ values from zero to 160, we expect our device to almost fully desaturate the RBCs.

**Fig 14:**
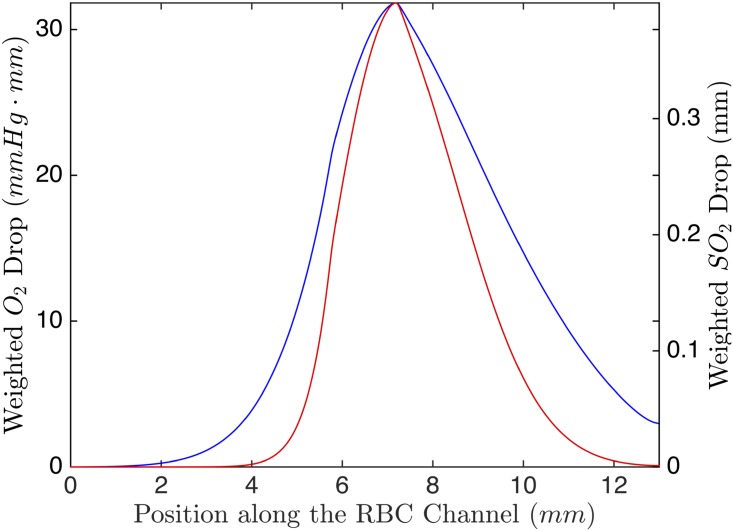
Simulation of Ideal Design. The ideal device was simulated with channel height of 0.5 mm and width of 0.3 mm, a 0.02 mm spin coat and a 1 mm long exchange window. This figure shows the weighted centreline drop in PO_2_ (blue) and the weighted centreline SO_2_ (red) as a function of position along the RBC channel. The black dashed line indicates the location of the exchange window.

The ideal device should have a low width to height ratio with a large area such that the width of the channel is much less than that of the exchange window. The device should have a spin-coat layer that is thin as possible, while still being able to withstand the flow pressure The exchange window should be long enough, but not so long that though it that the spin-coat layer sinks into the window. The hematocrit should be chosen to get a large enough signal from the ATP but small enough to not lose light from attenuation. The weighted centreline PO_2_ and SO_2_ drop are shown in Fig 14.

Microfluidic devices have become increasingly popular for use in biological studies due to their reduced sample consumption, relative low costs and length scales that are comparable to dimensions on the cellular level. Microfluidic devices are used in a wide range of biological applications including hemodynamics at the microvascular scale [32–34] and cell behaviour under shear stress [23, 35, 36]. Microfluidic devices can be used to establish gradients of small molecules [37–40]. Further, in recent years, microfluidic devices have been used to create micro-scale cell cultures that mimic *in vivo* micro-environments to study physiological tissue interactions [41–44]. Due to their vast applications in biological settings, microfluidic devices must be designed to meet the oxygen requirements of the study. Living cells are highly sensitive to the oxygen levels in their environment and may behave irregularly when exposed to unphysiological levels [45–47]. And since the results of our study demonstrate the crucial role that geometry can play in oxygen transport, it is an important consideration for the design of microfluidic channels. Our methods can also be applied to other gases and solutes as well as temperature.

Various studies have implemented mathematical models in order to validate and optimize microfluidic designs [13–15, 17, 18]. In these studies, the use of mathematical modelling was necessary for ensuring the proper distribution of oxygen in their application. Some of these studies considered only 2D models of their devices [13–15], while others used 3D models [17, 18]. In the studies that employed 2D models, 2D geometry was often sufficient to approximate the overall transport due to inherent symmetries in the device. In our application, a 3D geometry was crucial for two reasons. First, our device possessed two stream-wise directions (gas flow direction and RBC flow direction). Further a 2D model would not allow us to vary parameters that lied orthogonal to the plane that was being modelled.

In conclusion, although the 1D model provided important qualitative insights, the 3D model demonstrated that diffusion through the PDMS surrounding the RBC channel yielded unexpected relationships important to the design of the device. Thus the use of a 3D transport model is crucial for guiding our optimal design.
